# Effects of Diet—Exercise Interaction on Human Health across a Lifespan

**DOI:** 10.3390/nu15112520

**Published:** 2023-05-29

**Authors:** Ana Moradell, José Antonio Casajús, Luis A. Moreno, Germán Vicente-Rodríguez, Alba Gómez-Cabello

**Affiliations:** 1 GENUD (Growth, Exercise, NUtrition and Development) Research Group, Universidad de Zaragoza, 50009 Zaragoza, Spain; amoradell@unizar.es (A.M.); joseant@unizar.es (J.A.C.); lmoreno@unizar.es (L.A.M.); agomez@unizar.es (A.G.-C.); 2 Exercise and Health in Special Population Spanish Research Net (EXERNET), 50009, Zaragoza, Spain; 3 Department of Physiatry and Nursing, Faculty of Health, University of Zaragoza, 50009 Zaragoza, Spain; 4 Instituto Agroalimentario de Aragón (IA2), 50013 Zaragoza, Spain; 5 Instituto de Investigación Sanitaria Aragón (IIS Aragón), 50009 Zaragoza, Spain; 6 Centro de Investigación Biomédica en Red de Fisiopatología de la Obesidad y Nutrición (CIBEROBN), Instituto de Salud Carlos III, 28040 Madrid, Spain; 7 Department of Physiatry and Nursing, Faculty of Health and Sport Science FCSD, University of Zaragoza, 50009 Zaragoza, Spain; 8 Defense University Center, 50090 Zaragoza, Spain

The world is changing even faster than ever and has modified people’s lives [1,2]. Although globalization is an opportunity to develop technology, food, industry, health care advances and transport, it has changed people’s work, leisure, behaviours, and choices. These factors have also determined an increase in the prevalence of non-communicable diseases such as cardiovascular disease, diabetes, chronic respiratory disease, mental disorders and some cancers that appear across ages. Most of them are considered in the articles of this Special Issue. Noncommunicable diseases are attributed to 41 million deaths per year worldwide [3,4]. All these diseases are multifactorial, and lifestyle plays a fundamental role in their development; modifiable behaviours, such as diet and exercise, interact with their prevention and treatment [4]. Indeed, 1.8 million annual deaths have been attributed to excess salt/sodium intake; more than half of these 1.5 million have been attributable to alcohol use, and 830,000 deaths annually to insufficient physical activity [3]. However, although we have indications that the combination of factors is important, the question of how the beneficial effect of the interplay between exercise and diet can be enhanced is less well studied, probably due to greater methodological difficulty in study design and control. Therefore, this Special Issue addresses a complex and scientifically challenging topic. The studies featured in this Special Issue include research exploring the important interaction between diet and exercise across ages and different diseases, with a focus on the potential benefits and challenges.

From childhood to adolescence, this is a crucial period for establishing healthy lifestyle habits that can last a lifetime. Research has shown a healthy diet and regular physical activity in childhood can reduce the risk of chronic disease later in life [5,6,7]. A healthy diet, considered a diet rich in fruits, vegetables, whole grains, plant proteins, and dairy products, provides the essential nutrients needed for growth and development. However, achieving a healthy diet and regular exercise in childhood can be challenging, given the prevalence of unhealthy food consumption and sedentary lifestyles characterized by long periods of screen time. Some of the first large-scale studies associating dietary and movement behavioural patterns established a relationship between low levels of physical activity and sedentary behaviours with high sugared drinks and salted and sweetened snacks at pre-school ages [8] and then in children and adolescents [9,10]. This relationship increases the risk of developing obesity and other cardiovascular diseases, which may decrease the quality of life in this younger population. In this Special Issue, in the PASOS study, it is shown that a Mediterranean diet with higher fitness status can be related to higher levels of health-related quality of life, independent of the body mass index [11]. Therefore, these results highlight the importance of promoting healthy lifestyles to ensure the subject’s well-being, including an individual’s social, mental, and physical dimensions [12]. In this sense, combined interventions of nutrition and physical activity are the most promising [13,14,15].

Given the increased autonomy and independence of adolescents and young adults, the transition to this developmental stage is often accompanied by changes in diet and physical activity. It has been known for some years that this includes the increased consumption of unhealthy foods and a decrease in physical activity [16]. Peer pressure, busy schedules, and competing demands can make it difficult to prioritize healthy habits. These changes can lead to weight gain, obesity, and a higher risk of cardiovascular diseases later in life. It has been reported that more than one-third of people up to the age of sixteen in the European Union reported having a chronic health problem in 2021 [17]. Nonetheless, their desire to be independent makes them a target population to develop strategies that can improve their skills and healthy habits to be maintained in adulthood. In this regard, there is one article in this Special Issue that attempts to decrease cardiovascular diseases risk factors though an exercise and diet strategy [18]. Tang et al. performed a caloric restriction and rope-skipping program to improve cardiometabolic health in young adults [18]. They found that caloric restriction and both caloric restriction and rope skipping were effective in reducing body weight and obesity; however, only both together decreased the inflammation markers and metabolic profile. Thus, combining exercise and diet is a better strategy for cardiovascular prevention. Likewise, it is important to maintain this type of strategy over time to prevent the development of chronic conditions.

Maintaining caloric restriction could be difficult over time and in some specific cases. In this sense, the article in the framework of the BALANCE program focuses on the whole diet, recommending healthier dietary patterns to prevent the development of new cardiovascular events [11]. They observed an improvement in the dietary patterns with higher intakes of fruits and vegetables and a decrease in salted and sugared ultra-processed foods after an intervention. However, they did not observe a physical activity interaction between them. Further studies considering both dietary patterns and physical activity as lifestyle patterns are of interest, as a combination of them could probably increase benefits for health. In any case, it is essential to know how these main modifiable factors interact so that they can be considered in the field of nutrition or exercise, and exercise and dietary prescription can be more individualized, precise, and, therefore, efficient.

The evidence to date suggests that healthy dietary patterns reduce the risk of major diet-related chronic diseases, such as type 2 diabetes, cardiovascular disease and some cancers [7]. The study of dietary patterns has the difficulty of isolating unique foods or nutrients; however, this Special Issue includes an epidemiological article focusing on the prevalence of cancer and its relationship with coffee consumption and physical activity interaction [19]. They observed a lower odds ratio for most cancers in coffee consumers, probably due to their bioactive compounds but also higher odds of cancer in those with higher levels of physical activity. It is important that these results are interpreted with care and are adequately explained, as they can lead to misleading messages in the population. There are many advances and evidence of the independent benefits of exercise in different types of cancer [20]; the hypothesis remains that with adequate diet and physical exercise, cancer can be prevented, and treatment and recovery periods can be faced with greater guarantees. In fact, in this study [19], it has been speculated that this habit is achieved after the progression of the disease, as they are more conscious of their benefits. However, more research is needed as it seems the combined effect of both lifestyles may improve health, as a positive interaction was found between thyroid cancer and coffee consumers and physical activity practitioners [19]. 

As has been mentioned, evaluating single dietary components is complex. For this reason, during the last few years, analyses of dietary patterns and, more specifically, the Mediterranean diet have increased. The Mediterranean diet has been widely related to health outcomes [21]. It is considered in three of the nine articles in this Special Issue for different populations [22,23,24]. It is closely related to the quality of life in healthy school children [23], and moreover, it has also been related to self-perceptions of health in older adults [24]. Conde-Pipó et al. observed that a better physical self-concept was related to higher levels of Mediterranean diet adherence and higher levels of physical activity [24]. Furthermore, it is concerning that with age, there is a loss of these healthy patterns that are important for maintaining good health in older adults. Aging leads to physiological changes that compromise the independence of a person, increasing the risk of disease and leading to a high risk of falls and disability that could be aggravated if a person does not maintain healthy habits [25].

In this regard, aging is associated with multimorbidity. Metabolic syndrome components are abdominal obesity, hyperglycemia, high blood pressure, high levels of triglycerides and low levels of high-density lipoproteins cholesterol. Two studies in this Special Issue evaluate this pathology. Subías-Perie et al. indicates that a prevalence of around 30–40% do not observe a relationship between this condition and frailty [26]. However, they observed a low prevalence of abdominal obesity and hypertension in active participants, suggesting the beneficial effect of physical activity in the progression of this pathology [26]. Although individuals may experience health conditions as they age, Conde-Pipó et al. observed that older adults with metabolic syndrome had a higher health-related quality of life (HRQL) if they had better physical function [22]. Moreover, physical activity is not only important for the physical component of health but also for mental health. In this sense, the article written by Huang et al. describes the mediator effect of physical activity on reducing inflammation which has been associated with obesity and also depression [27]. 

Finally, taking into consideration polypharmacy, which is usually taken by older adults due to their multimorbidity, Navarrete-Villanueva et al. conducted a study to examine the relationship between obesity, fitness, and drug consumption [28]. The researchers categorized participants into different groups based on their obesity and fitness status. They found that those who were fitter with a lower fat mass consumed fewer drugs compared to those who had obesity or were less fit. This suggests that maintaining a healthy lifestyle and a balanced diet may lead to lower dependence on drugs. Apart from overall health and well-being benefits, these results also suggest an important step toward tools that can improve and make countries’ public health and health systems more sustainable.

To sum up, this Special Issue highlights how a healthy diet and regular exercise are essential for optimal health across a lifespan. The interaction between diet and exercise may have significant implications for health outcomes, including a reduced risk of cardiovascular and cancer diseases [18,19]. They improve mental health and well-being as well as maintaining physical function and independence at an older age [22,24,27]. However, achieving a healthy diet and regular exercise can be challenging, given the competing demands of modern life and age-related changes in health. Future research is needed for a better understanding of the interaction between diet and exercise on human health across lifespans and to develop individualized, precise, and, therefore, efficient interventions to promote healthy habits which can ensure adherence and maximize beneficial effects.
